# Growth Impairment of Small-Cell Cancer by Targeting Pro-Vasopressin with MAG-1 Antibody

**DOI:** 10.3389/fonc.2014.00016

**Published:** 2014-02-11

**Authors:** William G. North, Bernard Cole, Bonnie Akerman, Roy H. L. Pang

**Affiliations:** ^1^Department of Physiology and Neurobiology, Geisel Medical School at Dartmouth, Lebanon, NH, USA; ^2^Woomera Therapeutics Inc., Lebanon, NH, USA; ^3^Department of Mathematics and Statistics, The University of Vermont, Burlington, VT, USA

**Keywords:** small-cell lung cancer, targeted treatment, GRSA surface marker, pro-vasopressin, MAG-1 antibody, recurrent disease, STEPS concept

## Abstract

Previously we demonstrated that human small-cell lung cancer (SCLC) seems to universally express the vasopressin gene, and this leads to the presence of a cell surface marker representing the entire pro-hormone precursor. In this study, we show this marker can be targeted with MAG-1, a mouse monoclonal antibody against a C-terminal moiety on pro-vasopressin. *In vitro* targeting of cell lines derived from primary and recurrent disease demonstrates attachment of antibody to the cell surface followed by internalization. *In vivo* targeting with ^99^Tc-labeled Fab fragments of MAG-1 shows selective attachment to xenografts. *In vivo* treatment of tumors from classical cell line, NCI H345, with either ~1.65 mCi (~1.65 mg)/kg body weight (BW) of ^90^Yttrium-labeled MAG-1, or ~1.65 mg/kg BW native MAG-1, delivered every second day for 6 days produced similar reductions in the growth rate to ~50% (*p* < 0.03). When dosing with native MAG-1 was escalated to daily amounts of ~3.3 mg/kg BW over 16 days, tumor growth rates fell to ~33% of saline controls (*p* < 0.005). Examination of tumors treated with this higher dosing demonstrated the presence in several of extensive apoptosis. Normal tissues seemed to be unaffected. A larger dosage of MAG-1 (~6.6 mg/kg BW) given daily for 14 days was used to treat xenografts of the variant cell line NCI H82 representing recurrent disease. This treatment decreased the rate of increase in tumor size by half, and doubling time ~3-fold. Increases in cleaved PARP supported increased apoptosis with antibody treatment. We believe these data provide evidence that the growth rate of SCLC tumors can be extensively reduced by treatment with MAG-1 antibody, and that a humanized form of this antibody could, in future, be potentially used for targeting therapy onto recurrent SCLC in patients.

## Introduction

While some reduction in the incidence of lung cancer has occurred in recent times, there are still an expected 40,000 new cases of small-cell lung cancer (SCLC) per year in the United States. Present treatments of SCLC generally involve high-dose combination chemotherapy with or without radiation therapy (1–11). Although there is a high initial response rate to these treatments, and long-term survival in up to 10% of all cases (8, 9, 11), average life expectancy is increased by only 8–15 months. While about 80% of these newly diagnosed SCLC patients respond to chemotherapy, remission generally lasts only 3–6 months. Unfortunately, there is no effective therapy to treat recurrent disease because it is resistant to available approaches, including chemotherapies.

Small-cell lung cancer commonly expresses neuropeptides and one of us has advanced the *STEPS* concept of neuropeptide production by these tumors (12), which emphasizes process over origin and thus serves as a counter-balance to the *APUD* concept of Pierce (13). *STEPS* points to neuropeptides arising as part of the oncogenic process (rather than being present in cells from which tumors originate), and therefore reflects the now generally recognized “autocrine growth loop” involving peptides as a key survival strategy. We, and others, have demonstrated that vasopressin is one such neuropeptide (14–23). It is expressed by both primary and recurrence SCLC together with all four vasopressin receptors (19, 21), and has tumor growth modulating activities (18, 19, 23). Clinical evidence of ectopic production only occurs in 10–15% of cases, but expression of the vasopressin gene seems to be universal (20, 24–29). Tumor expression uniquely gives rise to some unprocessed precursor becoming a component of the plasma membrane, and this marker can be targeted with antibodies (30, 31). One such antibody is MAG-1, a mouse monoclonal antibody raised against the 18-mer C-terminal structure of the precursor (31). MAG-1 is of the IgG1 sub-class, and recognizes the marker in over 90% of SCLC tumors, and in all long-term cultures examined (20, 31, 32). MAG-1 has the ability to shrink and almost destroy both estrogen-responsive and triple-negative breast cancer xenografts (33). In the current study, we examined the effects of MAG-1 and ^90^Yttrium-labeled MAG-1, on the growth of human tumor xenografts derived from the classical cell line NCI H345, and the variant cell line, NCI H82, representing respectively, primary and recurrent disease.

## Materials and Methods

### Tumor cells

The human SCLC cell lines NCI H345 and NCI H82 were obtained from ATCC (American Type Culture Collection, Rockville, MD, USA) and maintained in DMEM medium (Mediatech, Inc., Herndon, VA, USA), containing 10% fetal bovine serum (Atlanta Biologicals) at 37°C, in an atmosphere of 5% CO_2_ with medium changes every 3 and 4 days. These cultures were grown in tissue culture flasks at densities from 1 to 5 × 10^5^ cells/ml. Since NCI H82 cells representing recurrent disease was the chief subject of investigations reported here, samples of the NCI H82 cells used here were sent back to ATCC at the completion of studies and verified by that body to indeed be representative of that long-term culture.

### Antibodies

The mouse monoclonal antibody, MAG-1, was generated against an 18-mer C-terminal segment of human pro-vasopressin (31–33), and MAG-1 is the subject of a patent by Woomera Therapeutics Inc. (34). MOPC21 is of the same IgG1 sub-class as MAG-1, but is a ubiquitous antibody produced by a mouse myeloma cell line (35). For this study, both antibodies were purified from culture by Protein A affinity chromatography (Bio-Xcell, Lebanon, NH, USA). For generation of radiolabeled form of MAG-1, the antibody was first chelated by reaction at pH 8.3 overnight with CHX-A″-DTPA reagent in 10-fold excess (36). DTPA-modified antibodies were then reacted with ^90^Yttrium chloride (Amersham >100 Ci/μmol, >500 mCi/ml) and ^90^Yttrium-labeled products isolated by Sephadex G-25 chromatography. Mouse polyclonal antibodies were generated against the 18-mer C-terminal segment of mouse pro-vasopressin (VQLAGTRESVDSAKPRVY), which has only 50% homology with the human C-terminus. The peptide was coupled to thyroglobulin using glutaraldehyde and used to immunize mice mixed 1:1 with Freund’s adjuvant. A preparation of rabbit polyclonal antibodies for measuring intact and 89-kDa cleaved PARP was purchased from Cell Signaling Technology (#9542, Danvers, MA, USA).

### Confocal microscopy

Small-cell lung cancer cells (NCI H345 and NCI H82) were seeded (10^4^) onto glass coverslips and allowed to recover for 24 h at 37°C. The cells were treated with a paraformaldehyde fixative buffer, permeabilized with 0.05% NP40 (Sigma-Aldrich Chemical Co.) and non-specific staining blocked with a gelatin buffer. They were then incubated with MAG-1 antibody (or MOPC antibody control) diluted in PBS containing 0.1% BSA, washed five times with buffer, and then incubated with Alexa Fluor 488 goat anti-mouse secondary antibody (Molecular Probes) for 1 h, following manufacturer recommendations. Finally, cells were washed (×5) with 1% BSA in PBS, the nuclei of the cells sometimes stained using DAPI, cells post-fixed with 4% paraformaldehyde and mounted in Vectashield (Vector Laboratories) on glass slides. Slides were visualized with an Olympus BX61WI fluorescence confocal microscope employing a Hamamatsu Orca-ER C4742-80 camera.

### Generation of NCI H345 and NCI H82 tumor xenografts

Male nu/nu mice 6–7 weeks of age were purchased from the NCI. NCI H345 and NCI H82 cells were trypsinized, concentrated into growing medium by centrifugation (4–5 × 10^7^ cells/ml) and injected subcutaneously in the lower right flank quadrant (1–2 × 10^7^ cells/animal) using a 1-ml syringe and 22-gage needle. Cells were allowed to generate tumor xenografts for 21 days (NCI H345), and 14 days (NCI H82), before the initiation of the studies. At these times, all of the mice receiving cells produced tumors that ranged in length from 0.5 to 0.75 cm. For the following 3 days, tumors were evaluated for tumor growth by measuring length, width, and depth with a micrometer (Electronic Digital Caliper, Fisher Scientific Inc.) and size expressed as the product of all three parameters. Measurements were performed on mice anesthetized with a mixture of isoflurane and oxygen (NCI 345), or without anesthesia (NCI H82).

An Fab fragment of MAG-1 was generated and isolated using Kit No. 448800 [Immunopure IgG1 Fab and F(ab′)2 preparation kit] from Pierce Chemical Co. (Rockford, IL, USA). This comprised adding MAG-1 antibody (1–5 mg/ml) in cysteine-reduced digestion buffer to a ficin-bound column and incubating for 3–5 h at 40°C. Products were passed through a Protein A column and the Fab fragment bound, then eluted and dialyzed.

CHX-A″-DTPA coupling reagent was used to generate a chelating group for the Fab product, and ^99^Tc, generated through SnCl2 reduction from ${\;}^{99}{\text{TcO}}_{4}^{-}$ added. For imaging, athymic nude mice received an intravenous injection of ^99^Tc-DTPA-CHX-A″-Fab MAG-1. Whole-body gamma camera scintigraphy of mice was performed at 10 min, 1, 4, and 24 h post-injection. Images were obtained with an E–Z hand-held gamma camera (Az-CA256AN) of the Anzai Medical Co., Ltd. (Japan) and data recorded on a dedicated computer.

### Treatment with antibodies

NCI H345 tumor-bearing mice were divided into two groups of four animals in one short-term treatment study on the effects of ^90^Yttrium-labeled antibodies, two groups of four animals in a second short-term treatment study on the effects of naked antibodies, and two groups of four animals in a long-term treatment study on the effects of naked antibodies. Animals were selected to provide a similar range in tumor size for each grouping. For each short-term study, group 1 comprised animals treated with four intra-peritoneal (i.p.) injections of 50 μl of saline vehicle given on days 0, 2, 4, and 6. Animals of group 2 received four i.p. injections of 50 μCi (initially 20 mCi/mg protein) of ^90^Yttrium-labeled MAG-1 (33) made up to 50 μg with naked MAG-1 carrier [~1.65 mg/kg body weight (BW)] in 50 μl saline. The amount of radiolabel and antibody employed was based on earlier studies by us and others (33, 37). For the second short-term study, groups 2 comprised treatments on days 0, 2, 4, and 6, with 50 μg/50 μl of naked MAG-1 (~1.65 mg/kg BW). Tumor volume was measured by micrometry and the BW of animals evaluated, daily, for 16 days. For the long-term study, animals were injected i.p. daily for 16 days with either saline vehicle (group 1), or with 100 μg/50 μl (~3 mg/kg BW) naked MAG-1 (group 2). Tumor size and animal BW were assessed daily over this period, and daily measurements of tumor volume and BW were extended for the MAG-1-treated group for an additional 20 days. Possible toxicity of treatment was measured by examining major organs (liver, kidneys) and tumors for necrotic changes at the end of the long-term study. For NCI H82 tumor-bearing mice, treatment was with 6.6 mg/kg BW of MAG-1 given daily for 14 days.

#### Assessment of apoptosis in NCI H82 tumors

A number of fine needle biopsies were performed on one MAG-1-treated (~3 mg/kg BW/day) and one saline-treated NCI H82 tumor in nu/nu mice, and tissues obtained were analyzed by Western Analysis for increases in cleaved (89 kDa) PARP as an index of apoptosis. Biopsies were taken using a 1″ 18-gage needle attached to a 1-ml syringe at times 0, 6, 24, 30, 48, and 54 h post-treatment from animals under isoflurane anesthesia. These biopsies routinely yield ~20 mg of tissue, and these tissues were spun free from needle and syringe and extracted for protein using SDS lysis buffer [2% SDS, 1% 2′-(*N*-cyclohexylamino)ethane sulfonic acid (CHES), 1% glycerol] in the presence of anti-protease cocktail (Sigma-Aldrich, St. Louis, MO, USA). Samples containing similar amounts of total protein (~20 μg) were separated on 12.5% gels by SDS-PAGE (25 mM Tris, 192 mM glycine, 0.1% SDS, pH 8.3), and then transferred onto Immobilon-P PVDF membrane (Millipore, Bedford, MA, USA) in the Tris-glycine buffer with 20% methanol added, using the Mini-Protean 3 system (Bio-Rad, Hercules, CA, USA). Membranes were blocked using 5% BSA in Tris-buffered saline with 0.1% Tween-20 (TBST), and relative amounts of both intact (116 kDa) and Caspase-cleaved (89 kDa) PARP were detected using a rabbit polyclonal antibody preparation from Cell Signaling Technology (Danvers, MA, USA, see above). After washing, a goat anti-rabbit HRP-conjugated secondary antibody preparation was employed (Santa Cruz Biotechnology, Santa Cruz, CA, USA) and detection was carried out using SuperSignal West Dura Extended Duration substrate (Pierce Chemical Co., Rockland, IL, USA) and imaged using a FluorChem 8900 imager (Alpha Innotech).

### Statistical analysis

Longitudinal growth data were evaluated by repeated measures of analysis of variance. The independent variables were factors for treatment group and time and for the interaction between treatment and time. In all cases, our analysis focused on comparing MAG-1 groups with the control groups. A two-sided *p*-value <0.05 was considered statistically significant.

## Results

### MAG-1 binds to the surface of SCLC cells

**Figure 1:**
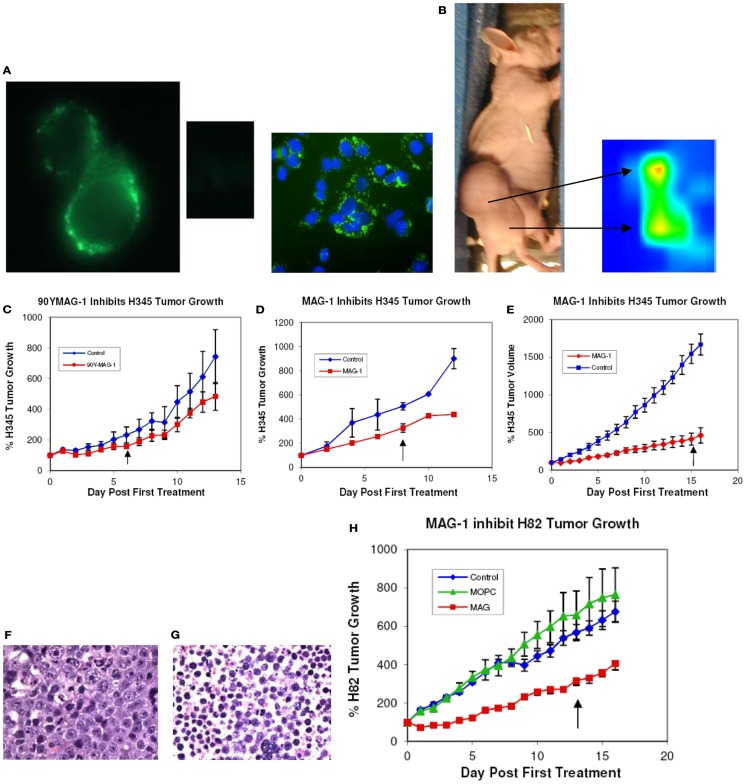
**(A)** Immunofluorescence and confocal imaging of NCI H82 cells with MAG-1 and MOPC21 (center insert); the blue staining of the confocal image is DAPI; **(B)** *in vivo* imaging of NCI H345 tumor in *nu/nu* mouse with ^99^Tc-DTPA-CHX-A″-Fab MAG-1 at 20 h after administration of label; **(C)** treatment of NCI H345 tumor in *nu/nu* mice with 50 μCi ^90^Y-DPTA-CHX-A″-MAG-1 (50 μg/30 g body weight total antibody) given alternate days (×4). Values expressed as % change in mean tumor size (±SEM) from day 0 (*n* = 4); **(D)** treatment of NCI H345 cells tumors in *nu/nu* mice *with* native MAG-1 (50 μg/g body weight) given alternate days (×4). Values expressed as % volume change (±SEM) from day 0 (*n* = 4); **(E)** treatment of NCI H345 cells tumors in *nu/nu* mice *with* native MAG-1 (100 μg/30 g body weight) given each day for 14 days. Values expressed as % change (±SEM) from day 0 (*n* = 8); **(F)** histology of control H345 tumor **(G)** and MAG-1-treated NCI H345 tumor; **(H)** treatment of NCI H82 tumor in *nu/nu* mice with native MAG-1, MOPC21 isotype antibody control, or saline vehicle. Antibody (200 μg/30 g body weight) was given daily for 14 days. Values expressed as % volume change for 14 days (*n* = 8) (↑, end of treatment).

Indirect fluorescence and confocal microscopy at low temperature revealed that MAG-1 antibody clearly binds to the surface, not only of NCI H345 cells, representing primary disease, but also of NCI H82 cells, representing recurrent SCLC. This is illustrated for NCI H82 cells in Figure 1A. At elevated temperatures, the antigen–antibody complex clearly relocated to inside of the cells as cytoplasmic clusters (data not shown). No fluorescence of cancer cells was obtained when MOPC21 antibody was used as negative control.

### MAG-1 targets SCLC xenografts in mice

Whole-body scintigraphy revealed that 20 h following i.p. delivery of ^99^Tc-CHX-A″-DPTA-Fab-MAG-1, radioactivity was located chiefly in the tumor. As indicated in Figure 1B, ^99^Tc-CHX-A″-DPTA-Fab-MAG-1 clearly imaged the H345 tumor in the tumor-bearing nude mouse with an estimated tumor to normal tissue ratio that ranged from 3.5 to >20.

### Short-term treatment with naked MAG-1 antibody as well as ^90^Yttrium-labeled MAG-1 decreases the rate of growth of NCI H345 SCLC tumors

Tumors treated over 6 days with either native MAG-1 (50 μg, *n* = 4) or ^90^Yttrium-labeled MAG-1 (50 μCi/μg protein with 50 μg MAG-1 carrier, *n* = 4) underwent a significant decrease in their rate of growth to ~50% of control rates during the treatment period. Since results with MAG-1 were similar with each short-treatment method, and since each treatment comprised the same total amount of antibody, we believe that most of the inhibitory effects seen by the end of the treatment with radiolabel were due to naked antibody. Nevertheless, while similar results were obtained with both treatments, there was still a marginally larger effect with radiolabeled antibody, which indicates that more pronounced effects could be obtained by targeting with sufficient amounts of MAG-1 modified with a lethal label. Results are shown in Figures 1C,D below, and are expressed as the percentage change in tumor size with treatment. During the period of observation, the tumors of both saline control groups increased in size by at least nine-times (>900%), while the ^90^Yttrium-labeled MAG-1 group and native MAG-1 group both increased by only ~4-times. In each study, there was a clear significant difference between saline and MAG-1 antibody treatments (*p* < 0.005 and *p* < 0.01).

### Extending and intensifying native MAG-1 treatment further impairs NCI H345 tumor growth

The influence on NCI H345 tumors of increasing the dose and frequency of native antibody to 100 μg/day (~3 mg/kg BW) and extending the treatment period from 6 to 16 days is shown in Figure 1E. This MAG-1 treatment of tumors (*n* = 4) caused a further reduction in growth rate to approximately one-quarter (~25%) of the growth rate and doubling time for saline-treated animals (*p* < 0.001). Saline-treated tumors (*n* = 4) showed rapid growth so that at 16 days, they were about 10 times their size at the start of the study.

### Treatment with native MAG-1 impairs NCI H82 tumor growth

NCI H82 is a variant cell line representing recurrent disease. Growth of tumor xenografts derived from this cell line was also impaired by treatment with native MAG-1 (*n* = 8) compared to saline treatment (*n* = 8) or treatment with MOPC21 (*n* = 8), a mouse immunoglobulin of the same sub-class (Figure 1H). The growth rate of MAG-1-treated tumors was about one-half of that of saline or MOPC21 treated tumors (*p* > 0.01), with a ~3-fold increase in the initial tumor doubling time.

### MAG-1 appears to increase NCI H82 tumor apoptosis

The amount of Caspase-cleaved PARP relative to total PARP, in tissues obtained through tumor needle biopsies, was employed as an index of apoptosis. From our initial studies comparing saline treated to MAG-1-treated tumor, apoptosis due to antibody increased following commencement of treatment from ~3% at 6 h, through ~40% at 24 h, to a maximum of ~55% at 30 h.

### MAG-1 antibody is selectively harmful to tumors

For both the short-term and long-term studies, fixed tissue examination revealed there were no structural or pathological changes induced in normal tissues by treatments with naked or ^90^Y-labeled MAG-1, while a substantial amount (30–50%) of necrosis was present in MAG-1-treated tumors compared with saline-treated tumors where there were little to no (<5%) necrotic changes evident. Examples of MAG-1-treated and saline-treated tumors of the extended study, stained with hematoxylin, are shown in Figures 1F,G.

### Antibody treatments have no apparent effects on animal well-being

Treatment with either naked MAG-1 or ^90^Y-labeled MAG-1 had no apparent effect on the well-being of tumor-bearing animals as judged by their behavior and BWs. There were no discernible differences in BW changes of the period of observation between any of the groups. There was no observable pathology of the livers and kidney from short-term MAG-1 and ^90^Y-labeled MAG-1-treated animals. In addition, histological evaluation of organ tissues such as the liver and kidneys revealed there was no damage to these tissues by extensive treatment with native MAG-1. Since there is only 50% homology between the mouse and human pro-vasopressin 18-mer C-terminus, we generated polyclonal antibodies in mice against the mouse peptide to look for possible negative consequences. Two of three mice immunized with the C-terminal 18-mer of mouse pro-vasopressin produced high titer antibodies (>1:10,000) against this peptide. However, the presence of these polyclonal antibodies did not appear to disturb in any way the physiology or behavior of animals.

## Discussion

The data presented here illustrates that a monoclonal antibody, MAG-1, can target tumor xenografts of SCLC in nu/nu mice, and significantly impair the growth of such tumors. MAG-1 is a mouse IgG1 immunoglobulin, and was raised against the C-terminal 18 residues of human pro-vasopressin earlier shown by us to be a surface marker of most, or all, small-cell tumors (20, 31, 32). Targeting was directed to the surface of tumor cells as shown by confocal microscopy, and targeting of tumor xenografts seemed to be specific from ^99m^Technetium-labeled Fab imaging studies. The fourfold reduction in growth rate by MAG-1 antibody at ~3 mg/kg BW, of xenografts derived from a classical cell line representing primary SCLC tumors strongly suggests that a humanized version of MAG-1 could be used in conjunction with chemotherapy to more effectively treat the primary form of this disease in patients. Such antibody–chemotherapy combinations have proved to be very effective in breast cancer with Herceptin where disease recurrence was reduced by half of that occurring following chemotherapy alone (38). The marker targeted by MAG-1 antibody is most importantly present on recurrent disease for which there is currently no effective treatment. While treatment with native MAG-1 seems to be more effective on tumors derived from the classical cell line NCI H345 representing primary disease, it still has a significant effect on tumors derived from the variant cell line NCI H82, obtained from a pleural effusion and representing recurrent SCLC (39, 40). This effect was to increase tumor doubling time three to fourfold over controls (*p* < 0.01). Since a dose response effect of the antibody on growth, was seemingly demonstrated at least for NCI H345 tumors, it is possible that higher amounts of antibody than used here could have even greater impacts on the growth of SCLC tumors representing both primary and recurrent disease. Clearly, significant apoptosis was demonstrated by the pathology in fixed tissues of residual tumors from treatment with higher amounts of MAG-1 antibody. In the case of NCI H82 tumor, antibody-induced apoptosis in initial studies was also demonstrated by increased PARP cleavage. Although the mechanism for the effectiveness of MAG-1 is still not known beyond this apoptosis, the marker-antibody complex was shown to become internalized.

While most of the reduction in tumor growth observed was by native MAG-1 antibody alone, there was however, a marginally greater effect seen when MAG-1 was attached to ^90^Yttrium. This finding indicates that this and other modified forms of the antibody might have an additional impact in potential future targeted treatments.

Native MAG-1 has already been shown by us to prevent growth and shrink xenografts of estrogen-responsive and triple-negative human breast cancer (33). Effects of MAG-1 treatments were more dramatic than those shown here for SCLC, and were accomplished with the smaller amounts of antibody than used for treatment of SCLC (~1.5 mg/kg BW). Additionally, most treated breast tumors failed to rebound after treatment stopped, while in most cases the major negative effects of MAG-1 on SCLC growth occur during an early stage of treatment. Tumors representing recurrent disease then typically increased their growth rate to one exhibited by controls treated by saline vehicle or with ubiquitous MOPC1 antibody. Nevertheless, the initial influences on growth reduction observed in this study are substantial given that there is currently no successful therapy for recurrent SCLC.

This raises the possibility of a humanized form of the MAG-1 antibody to be used in the development of therapies against this recurrent disease. The actions of MAG-1 antibody are particularly relevant since we have previously shown the surface antigen on tumors recognized by the antibody can be effectively targeted in patients (30). This antibody targeting of pro-vasopressin was found to be specific for tumors, and it was later shown that MAG-1 did not react with any of an array representing 66 different normal human tissues (31). MAG-1 in mice is also tumor selective and treatment with this antibody had no deleterious effects on normal tissues of these animals. However, because this latter finding could have been, in part due to a failure of MAG-1 to recognize mouse pro-vasopressin, we generated polyclonal antibodies in mice against the C-terminal region of the mouse molecule. The presence of high titers of these polyclonal antibodies (effective in radioimmunoassay at serum dilution >1:40,000) failed to affect mice in any way, including an absence of diabetes insipidus.

## Author Contributions

William G. North drafted the manuscript and was the chief architect of this study. He generated the antibody, prepared derivatives of the antibody, injected animals with cells to produce xenografts, and performed imaging and treatment studies with animals. Bernard Cole was responsible for the biostatistical evaluation of the data. Bonnie Akerman performed confocal imaging studies and Roy H. L. Pang prepared cells and assisted in treatment studies.

## Conflict of Interest Statement

Both William G. North and Roy H. L. Pang are associated with Woomera Therapeutics Inc., which has a commercial interest in the antibody MAG-1. Bernard Cole and Bonnie Akerman have no such conflict of interest.

## References

[1] Johnson BE Grayson J Makuch RW Linnoila RI Anderson MJ Cohen MH Ten-year survival of patients with small cell lung cancer treated with combination chemotherapy with or without irradiation. J Clin Oncol (1990) 8:396–401 2155310 10.1200/JCO.1990.8.3.396

[2] Wampler GL Heim WJ Ellison NM Ahlgren JD Fryer JG Comparison of cyclophosphamide, doxorubicin, and vincristine with alternating regimen of methotrexate, etoposide, and cisplatin/cyclophosphamide, doxorubicin, and vincristine in the treatment of extensive-disease small cell lung carcinoma: a Mid-Atlantic Oncology Program Study. J Clin Oncol (1991) 9:1438–45 1649265 10.1200/JCO.1991.9.8.1438

[3] Cook RM Miller YE Bunn PA Small cell lung cancer: etiology, biology, clinical features, staging, and treatment. Curr Probl Cancer (1993) 17:71–141 10.1016/0147-0272(93)90010-Y 8395998

[4] van Zandwijk N Are we moving towards continuous treatment in small cell lung cancer (SCLC). Anticancer Res (1994) 14:309–12 8166473

[5] Moro D Small cell lung cancer: patients surviving longer than thirty months. Anticancer Res (1994) 14:301–4 8166471

[6] Maurer LH Herndon JE II Hollis DR Aisner J Carey RW Skarin AT Randomized trial of chemotherapy and radiation therapy with and without warfarin for limited-stage small cell lung cancer: a Cancer and Leukemia Group B study. J Clin Oncol (1997) 15:3378–87 9363869 10.1200/JCO.1997.15.11.3378

[7] Sandler AB Current management of small cell lung cancer. Semin Oncol (1997) 24:463–76 9280226

[8] Zangemeister-Wittke U Stahel RA Novel approaches to the treatment of small cell lung cancer. Cell Mol Life Sci (1999) 55:1585–98 10.1007/s000180050398 10526576 PMC11146849

[9] Ciomber KK Rocha Lima CM Management of small cell lung cancer. Curr Treat Options Oncol (2006) 7:59–68 10.1007/s11864-006-0032-7 16343369

[10] Azim HA Ganti AK Treatment options for relapsed small-cell lung cancer. Anti-cancer Drugs (2007) 18:255–61 10.1097/CAD.0b013e328011a547 17264756

[11] Hann CL Rudin CM Management of small-cell lung cancer: incremental changes but hope for the future. Oncology (2008) 22:1486–92 19133604 PMC4124612

[12] North WG Gene regulation of vasopressin and vasopressin receptors in cancer. Exp Physiol (2000) 85S:27–40 10.1111/j.1469-445X.2000.tb00005.x 10795904

[13] Pierce AG The cytochemistry and ultrastructure of polypeptide hormone producing cells of the APUD series, and the embryologic, physiologic, and pathologic implications of the concept. J Histochem Cytochem (1969) 17:303–13 10.1177/17.5.303 4143745

[14] Woll PJ Rozengurt E Neuropeptides as growth regulators. Br Med Bull (1989) 45:492–505 2557117 10.1093/oxfordjournals.bmb.a072337

[15] Friedmann AS Memoli VA North WG Vasopressin and oxytocin production by non-neuroendocrine lung carcinomas: an apparent low incidence of gene expression. Cancer Lett (1993) 75:79–85 10.1016/0304-3835(93)90191-B 8293425

[16] North WG Yu X-M Forms of neurohypophysial peptides generated by tumors, and factors regulating their expression. Regul Pept (1993) 45:209–16 10.1016/0167-0115(93)90208-P 8390078

[17] North WG Yu X-M Vasopressin mRNA and neurophysin-related cell-surface antigen (NRSA) in small-cell carcinoma. Peptides (1993) 14:303–7 10.1016/0196-9781(93)90045-I 8387189

[18] Bunn PA Effects of neuropeptide analogues on calcium flux and proliferation in lung cancer cell lines. Cancer Res (1994) 54:3602–10 7516822

[19] Fay MJ Friedmann AS Yu X North WG Vasopressin and vasopressin receptor immunoreactivity in small-cell lung carcinoma (SCCL) cell lines: disruption in the activation cascade of V_1a_-receptors in variant SCCL. Cancer Lett (1994) 82:167–74 10.1016/0304-3835(94)90007-8 8050087

[20] Friedmann AS Malott KA Memoli VA Pai SI Yu X-M North WG Products of vasopressin gene expression in small cell carcinoma of the lung. Br J Cancer (1994) 69:260–3 10.1038/bjc.1994.49 8297723 PMC1968694

[21] North WG Fay MJ Longo KA Du J Expression of all known vasopressin receptor subtypes by small cell tumors implies a multifaceted role for this neuropeptide. Cancer Res (1998) 58:1866–71 9581826

[22] Coulson JM Stanley J Woll PJ Tumour-specific arginine vasopressin promoter activation in small-cell lung cancer. Br J Cancer (1999) 80:1935–44 10.1038/sj.bjc.6690623 10471042 PMC2374275

[23] Piqueux C Hagelstein MT Keegan B Legros V North WG Vasopressin and oxytocin mitogen transduction pathway in small cell lung cancer cells. Endocr Relat Cancer (2004) 4:871–5 10.1677/erc.1.00803 15613460

[24] North WG Maurer LH Valtin H O’Donnell JF Human neurophysins as potential tumor markers for small cell carcinoma of the lung: application of specific radioimmunoassays for vasopressin-associated and oxytocin-associated neurophysins. J Clin Endocrinol Metab (1980) 51:892–6 10.1210/jcem-51-4-892 6252226

[25] North WG Ware J Chahinian AP Perry M O’Donnell J Maurer LH Clinical evaluation of the neurophysins as tumor markers in small cell lung cancer. Recent Results Cancer Res (1985) 99:187–93 2999918 10.1007/978-3-642-82533-0_21

[26] North WG Ware J Maurer LH Chahinian AP Perry M Neurophysins as tumor markers for small cell carcinoma of the lung: a cancer and leukemia group B evaluation. Cancer (1988) 62:1343–7 10.1002/1097-0142(19881001)62:7<1343::AID-CNCR2820620717>3.0.CO;2-H 2843278

[27] Legros JJ Geenen V Carvelli T Martens H Andre M Corhay JL Neurophysins as markers of vasopressin and oxytocin release. A study in carcinoma of the lung. Horm Res (1990) 34:151–5 10.1159/000181815 1966564

[28] North WG Neuropeptide production by small cell carcinoma: vasopressin and oxytocin as plasma markers of disease. J Clin Endocrinol Metab (1991) 73:1316–20 10.1210/jcem-73-6-1316 1659583

[29] North WG Friedmann AS Yu X-M Tumor biosynthesis of vasopressin and oxytocin. Ann N Y Acad Sci (1993) 689:107–21 10.1111/j.1749-6632.1993.tb55541.x 8396864

[30] North WG Hirsh V Lisbona R Schulz J Cooper B Imaging of small cell carcinoma using ^131^I-labeled antibodies to vasopressin-associated human neurophysin (VP-HNP). Nucl Med Commun (1989) 10:643–51 10.1097/00006231-198909000-00003 2559381

[31] North WG Memoli VA Keegan BP Immunohistochemical detection of NRSA on small cell lung cancer with a monoclonal antibody (MAG-1) that recognizes the carboxyl terminus of provasopressin. Appl Immunohistochem Mol Morphol (2005) 13:363–6 10.1097/01.pai.0000149939.12822.ee 16280667

[32] Keegan BP Memoli VA North WG Targeting the neurophysin-related cell surface antigen (NRSA) on SCLC cells using a monoclonal antibody against the glycopeptide region (MAG-1) of provasopressin. Mol Cancer Ther (2002) 1:1153–9 12479696

[33] North WG Pang RH Gao G Memoli VA Cole BF Native MAG-1 Antibody almost destroys human breast tumor xenografts. Breast Cancer Res Treat (2010) 122:307–14 10.1007/s10549-010-1009-6 20625819 PMC4521588

[34] North WG Keegan B Oligino L Compositions and Uses Thereof for Identifying and Targeting Provasopressin-Expressing Cancer Cells. US patent application #10/521,091 (2005).

[35] Schubert D Immunoglobulin assembly in a mouse myeloma. Proc Natl Acad Sci U S A (1968) 60:683–90 10.1073/pnas.60.2.683 4178281 PMC225100

[36] Adams GP Shaller CC Dadachova E Simmons HH Horak EM Tesfaye A A single treatment of yttrium-90-labeled CHX-A″-C6.5 diabody inhibits the growth of established human tumor xenografts in immunodeficient mice. Cancer Res (2004) 64:6200–6 10.1158/0008-5472.CAN-03-2382 15342405

[37] Stein R Chen S Goldenberg DM Advantage of yttrium-90-labeled over iodine-131-labeled monoclonal antibodies in the treatment of a human lung carcinoma xenograft. Cancer (1997) 80:2636–41 10.1002/(SICI)1097-0142(19971215)80:12+<2636::AID-CNCR39>3.0.CO;2-B 9406718

[38] Perez EA Suman VJ Davidson NE Gralow JR Kaufman PA Visscher DW Sequential versus concurrent trastuzumab in adjuvant chemotherapy for breast cancer. J Clin Oncol (2011) 29:4491–7 10.1200/JCO.2011.36.7045 22042958 PMC3236650

[39] Carney DN Gazdar AF Bepler G Guccion JG Marangos PJ Moody TW Establishment and identification of small cell lung cancer cell lines having classic and variant features. Cancer Res (1985) 45:2913–23 2985257

[40] Gazdar AF Carney DN Nau MM Minna JD Characterization of variant subclasses of cell lines derived from small cell lung cancer having distinctive biochemical, morphological, and growth properties. Cancer Res (1985) 45:2924–30 2985258

